# Tropical cyclone-driven storm surge and wave database for the US North Atlantic and Gulf coastlines

**DOI:** 10.1038/s41597-026-07635-z

**Published:** 2026-06-26

**Authors:** Mithun Deb, Karthik Balaguru, Julian Rice, Tim McPherson, W. Brent Daniel

**Affiliations:** 1 Coastal Sciences Division, Energy and Environment Directorate, Pacific Northwest National Laboratory, Seattle, WA 98109 USA; 2 Energy and Environment Directorate, Pacific Northwest National Laboratory, Richland, WA 99354 USA; 3 Present Address: Environmental Hydrology and Design, Environmental Science Associates, San Francisco, CA 94105 USA

## Abstract

Historical datasets of tropical cyclone-driven storm surge and waves at moderate coastal resolution are scarce, limiting coastal hazard analysis and AI/ML-based surrogate model development where field observations remain sparse. We present a publicly available hindcast database of surge and wave conditions for 232 U.S. landfalling and impactful storms (1981-2021). We applied the coupled ADCIRC+SWAN system across the entire U.S. North Atlantic and Gulf coastline on a coastal-refined unstructured mesh achieving practical nearshore resolution (~100 − 500 m) for computational feasibility. We forced simulations with parametric wind fields from the Generalized Asymmetric Holland Model fitted to NOAA best-track data. For each event, we provide hourly NetCDF files containing water-surface elevation, significant wave height, and peak wave period. Users can apply these fields as boundary conditions for higher-resolution local models, train ML model predictors, and conduct coastwide extreme-value analyses. We validated simulations against numerous NOAA tide gauges and NDBC buoys, demonstrating robust water level skill with documented wave biases. This comprehensive basin-scale database enables coastal flood hazard assessment across multiple decades of historical storm surge events.

## Background & Summary

Regions along the U.S. North Atlantic and Gulf coast face increased exposure to tropical cyclone (TC) hazards, mainly from storm surge and waves that drive coastal flooding and erosion^1–3^. The risks are amplified by rising sea levels and the rapidly growing coastal development, threatening people, critical infrastructure, and natural ecosystems^4,5^. Nearly $1 trillion of property and more than 40% of the U.S. population live in coastal counties^6^. Recent major hurricanes Harvey (2017), Michael (2018), and Ian (2022), caused an estimated $155.0B, $25.5B, and $116.3B worth of total damage, respectively^7^.

Observational networks (tide gauges and wave buoys) maintained by the National Oceanic and Atmospheric Administration (NOAA) provide essential field datasets but remain spatially and temporally limited^8^, especially in the nearshore, where buoys are sparse and can fail during extreme conditions. These data gaps restrict coastwide hazard analyses (e.g., extreme value analysis) and limit the sample to train data-driven statistical or AI/ML models. In particular, publicly available historical data records combining surge and wave properties at a practical, moderate coastal resolution (roughly 100-500 m in the nearshore) along the entire U.S. coastline are scarce.

Traditionally, the coastal modeling community uses physics-based hydrodynamic and wave models like ADCIRC^9,10^, FVCOM^11^, Delft3D^12^, and SWAN^13^ to construct surge and wave fields for hurricane events. Coupled surge-wave frameworks like ADCIRC+SWAN more effectively capture two-way interactions, such as wave radiation stress, wave setup, and total water level, which lead to better predictions of peak surge levels and currents^14–16^. These improvements are crucial for realistic flood assessments. However, conducting basin-scale coupled hindcasting at a practical nearshore resolution is computationally demanding and often applied unevenly in regions where storms affect the area of interest, even achieving less than 20 meter resolution^15,17^.

**Fig. 1:**
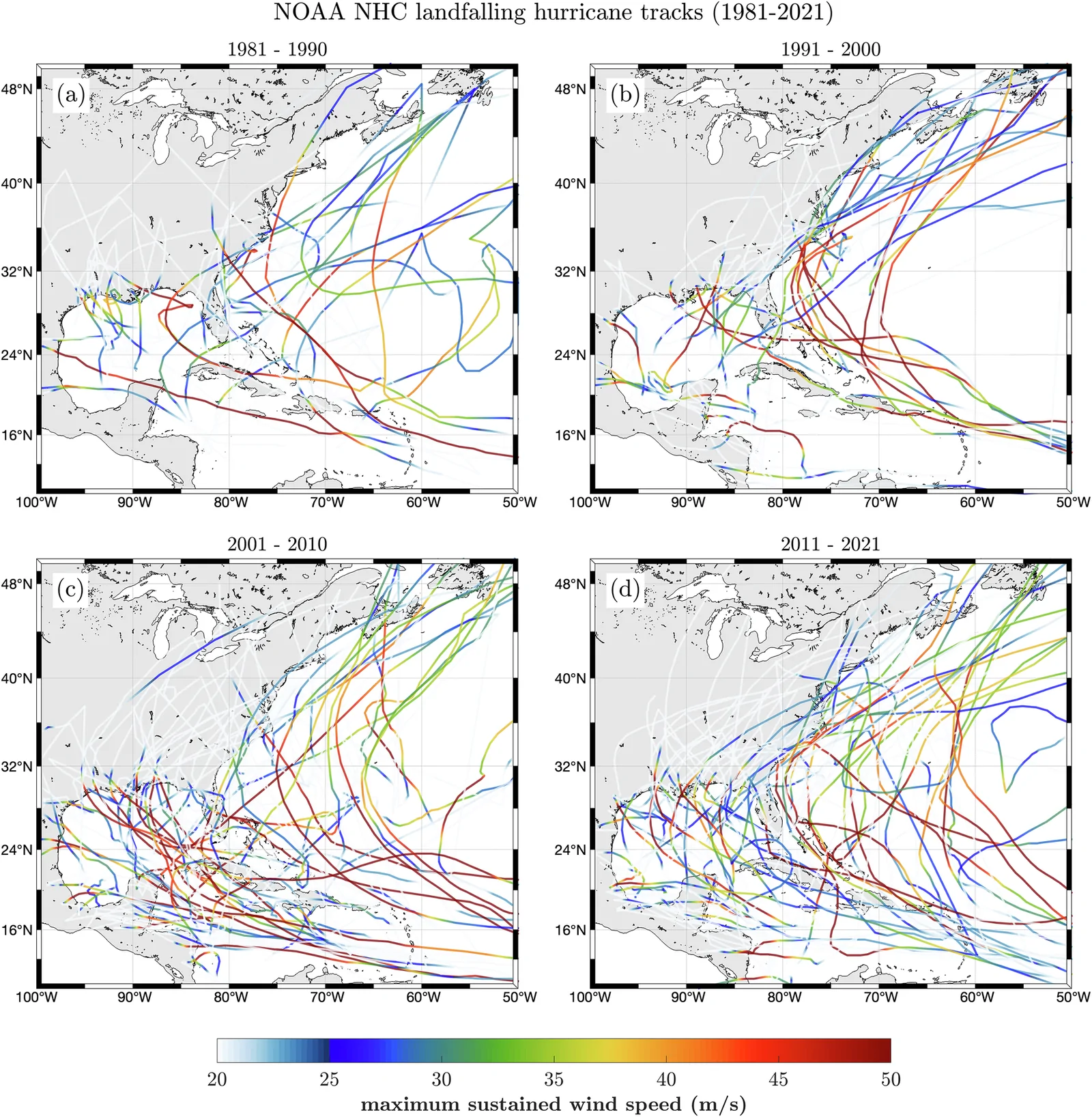
Tracks of the 232 tropical cyclones that made landfall in the U.S. from 1981 to 2021. The data is sourced from the NOAA National Hurricane Center’s (NHC) official archive^20^, showing the paths of these impactful events. Track color indicates the *maximum sustained wind speed* (1-minute average, as recorded in the NHC best-track data) at each best-track position.

We address these limitations by producing a basin-scale hindcast database for 232 U.S. landfalling and impactful storms (1981-2021), shown in Fig. 1, using the coupled ADCIRC+SWAN framework on a coastal-refined unstructured mesh that reaches a nearshore resolution of 100-500 m. We ran the model simulations with the quality-controlled National Hurricane Center (NHC) best-track data and provided hourly outputs of water surface elevation, significant wave height, and peak wave period in NetCDF format.

This dataset fills a critical need for: (1) coastwide extreme-value and trend analyses where observations are sparse; (2) training and benchmarking of machine-learning-based surge and wave models; and (3) providing ready boundary/forcing conditions for higher-resolution local and regional hydrodynamic and wave simulations. We validated the simulations against numerous NOAA tide gauges^18^ and National Data Buoy Center (NDBC) wave buoys^19^ for various TC events and regions to quantify model skill and systematic biases. We also provided users with guidance on the data usage and limitations.

By delivering a consistent, quality-controlled hindcast archive at a practical resolution across four decades, we provide a valuable basin-scale resource that supports coastal flood assessment, infrastructure vulnerability studies, and resilience planning for U.S. Atlantic and Gulf coasts.

## Methods

We developed the storm surge and wave database through a five-step process: (1) selecting and processing historical tropical cyclone (TC) track data that affected U.S. coastlines; (2) configuring and executing the coupled SWAN+ADCIRC modeling system; (3) extensively collecting water level, wave height, and wave period data from NOAA/NDBC field gauges; (4) validating the model and estimating error metrics; and (5) post-processing and archiving the model results.

### Historical Hurricane Data and Storm Selection

The basis of this database is the historical hurricane track record obtained from the NOAA National Hurricane Center’s (NHC) official archive^20^. From this comprehensive record, we selected 232 impactful hurricane events that made landfall in the U.S. between 1981 and 2021 (Fig. 1). We chose this specific timeframe because storms occurring before 1981 typically lack essential structural information, especially the radius of maximum winds (Rmax), which is necessary for accurately reconstructing wind fields. The events chosen in this study include all landfalling hurricanes and other significant TCs that affected the U.S. Atlantic and Gulf coasts during these four decades. The selection criteria focused on factors such as where the storms made landfall and their proximity to the coast.

### Numerical Modeling Framework

The Advanced Circulation Model (ADCIRC) 2D is an unstructured grid, depth-integrated, and barotropic hydrodynamic model that solves the shallow-water equations using a continuous Galerkin finite-element method^9,10^. The model employs a Generalized Wave Continuity Equation (GWCE) to determine the free-surface elevation and the vertically integrated momentum equations for currents. Detailed equations can be found in Luettich *et al*.^9^ and Westerink *et al*.^10^. An unstructured grid increases computational efficiency by providing more geometric flexibility and allows localized refinement in complex nearshore regions such as inlets, estuaries, and floodplains. ADCIRC has been extensively implemented and validated for hurricane surge hindcasts and forecasts in the U.S., with applications ranging from basin to channel scales^5,14,16,21^. It has also been utilized internationally for flood risk assessments across various coastal settings with differing topo-bathymetry and storm climates^22–25^.

In large domain simulations, resolving the phase and processes of wind waves is often impractical due to the potentially small time and length scales involved^14^. The Simulating WAves Nearshore model (SWAN) is a third-generation spectral wave model that solves the wave action balance equation in geographic-spectral space^26^, representing a phase-averaged wave field. Source and sink terms represent wind input, whitecapping, depth-induced breaking, bottom friction, and nonlinear interactions. SWAN’s initial implementation utilized finite-difference numerics on structured grids, but more recent versions also accommodate unstructured triangular meshes^13^.

The ADCIRC+SWAN coupled system facilitates two-way interaction between currents and waves, leading to more physically accurate total water levels than standalone models^15^. In this framework, ADCIRC provides time-varying water levels and depth-averaged currents to SWAN, which then models the wave propagation, shoaling, and breaking. SWAN returns wave radiation stress to ADCIRC, which is incorporated into the momentum equations to account for wave setup and wave-driven currents. This coupling improved the accuracy of peak coastal water levels and currents during several major hurricanes, including Gustav, Ike, and Sandy^16,27^. It is now widely utilized by U.S. and international agencies for surge and wave hindcasts, risk analyses, and the development of forecast systems^28,29^.

### Model Unstructured Grid

**Fig. 2:**
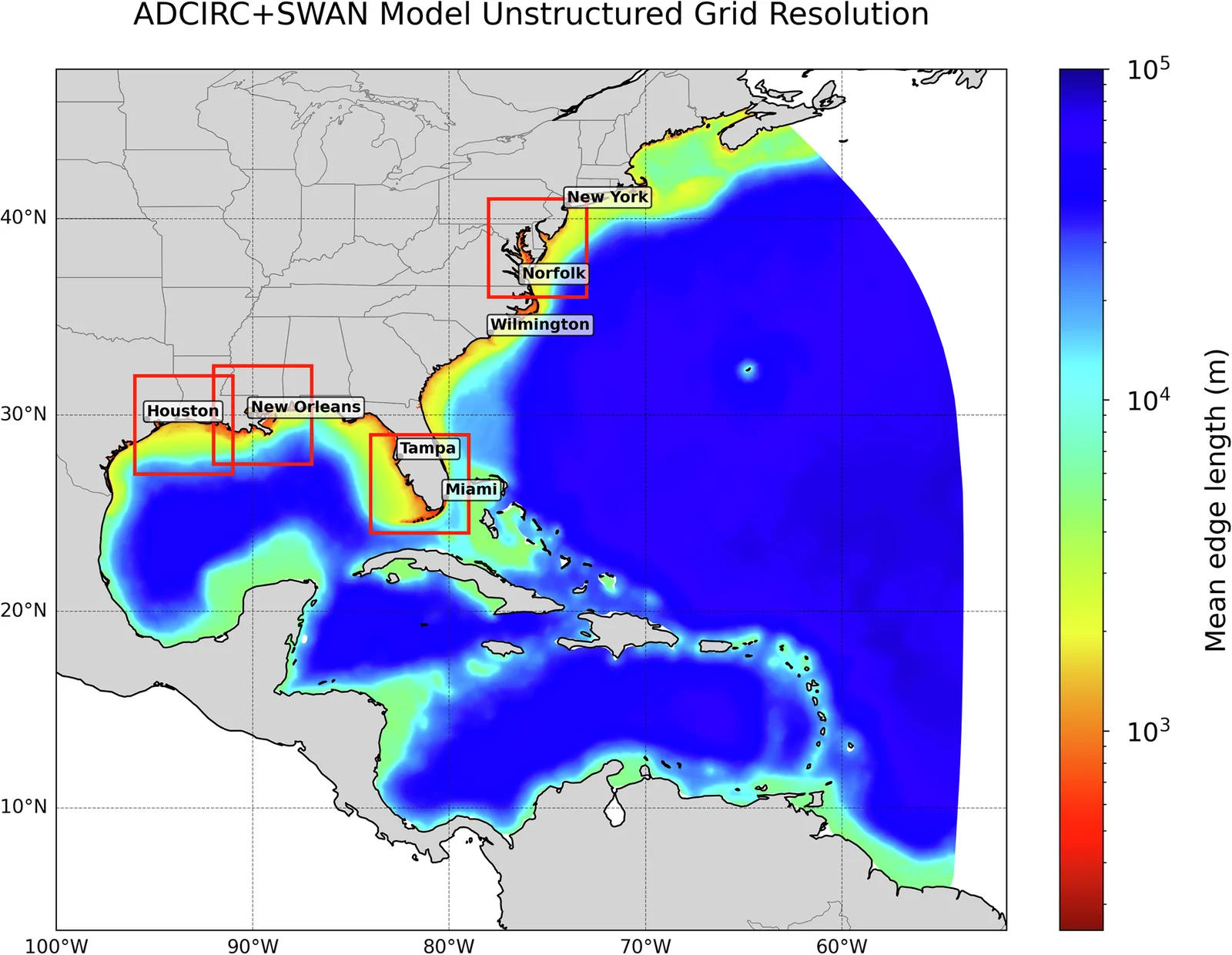
ADCIRC+SWAN model domain and unstructured grid resolution. Red polygons indicate four U.S. coastal regions frequently affected by landfalling hurricanes and associated coastal hazards.

We generated a custom, high-resolution unstructured grid for this study that covers the U.S. Atlantic and Gulf coastlines, extending into the western North Atlantic Ocean and the Caribbean Sea. The mesh resolution is under 1 kilometer along all coastal areas, with refinement down to about 200 meters in critical regions, frequently affected by landfalling hurricane surges (polygons shown in Fig. 2).

For grid generation and model bathymetry, we utilized the bathymetry data from the high-resolution EC2015 grid developed by Szpilka *et al*.^30^ to provide an updated tidal database for the U.S. Atlantic and Gulf coastlines. Szpilka *et al*.^30^ developed the mesh bathymetry by assimilating data from multiple sources, including the Earth Topography One Arc-Minute Global Relief Model (ETOPO1), National Geophysical Data Center (NGDC) coastal relief models, NOAA hydrographic surveys, and U.S. Army Corps of Engineers (USACE) navigation channel surveys. The EC2015 grid represents the latest evolution of the Western North Atlantic Tidal (WNAT) database series. It began with EC1991, which contained approximately 20,000 nodes at a resolution of 7 km. Then EC1995 was refined to about 5 km using hydrographic survey data from NOAA/NOS. EC2001 increased the resolution to include 250,000 nodes, achieving a mesh size of 200 m in the Mississippi River Delta and 500 m near New Orleans. The EC2015 update further improved the detail and coverage, reaching over 2 million nodes and 3.8 million elements, with typical resolutions of 250-500 m along most coasts and as fine as 13 m in narrow inlets, such as those in Puerto Rico and Long Island Sound. Szpilka *et al*.^30^ also updated the offshore bathymetry using the SRTM30_PLUS model^31^.

We directly imported the bathymetry data from Szpilka *et al*.^30^, and our ADCIRC model grid bathymetry is referenced to a vertical datum known as Model Zero (MZ), initially developed as part of NOAA’s VDatum project^30^. The MZ datum provides a unified vertical reference surface across the model domain by harmonizing bathymetric data from various sources and datums. MZ is an approximate representation of Mean Sea Level (MSL), as it closely aligns with local MSL in open ocean areas. However, deviations may occur from place to place in complex nearshore regions due to non-linear processes.

**Fig. 3:**
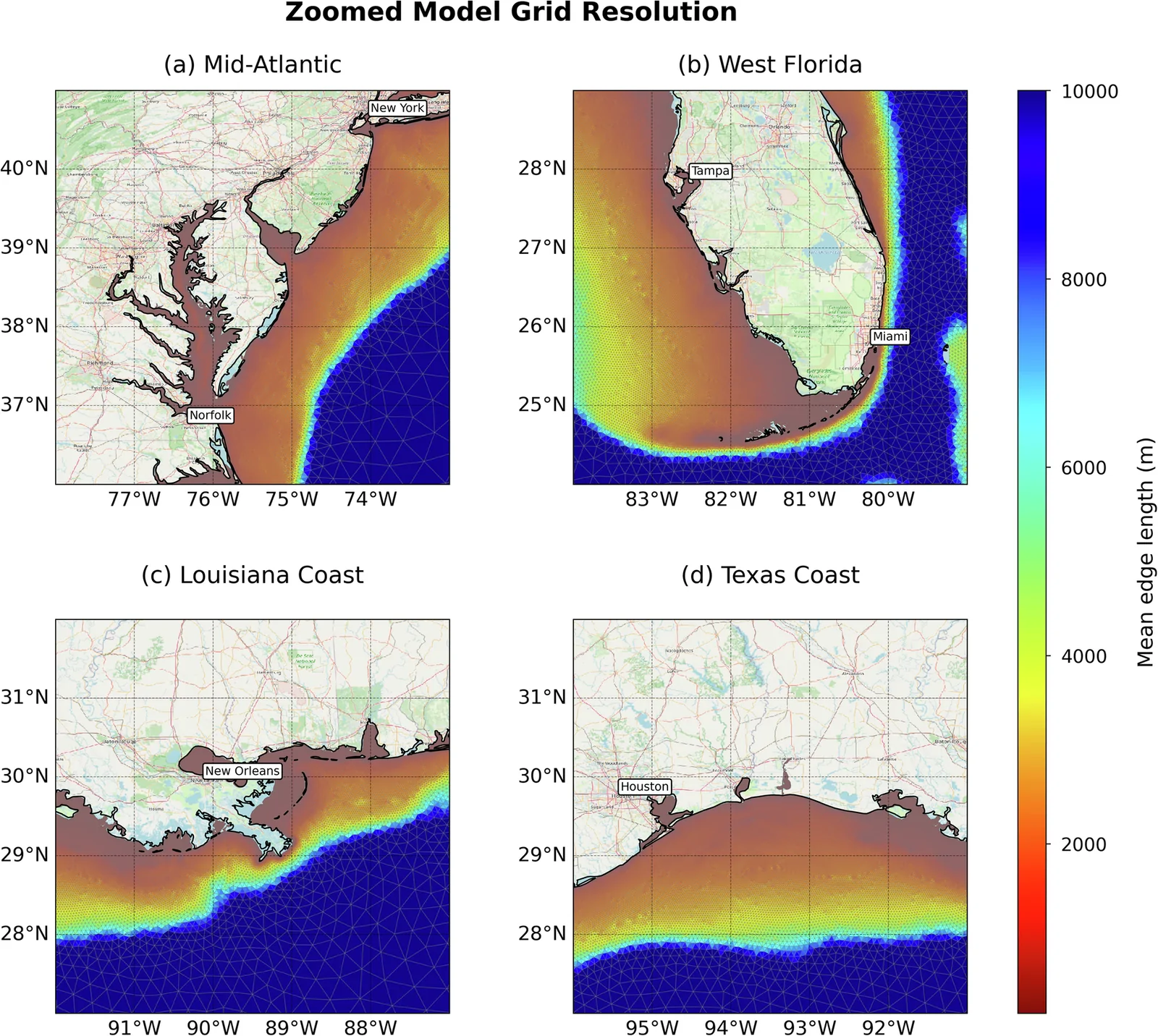
Inset views of the model grid resolution corresponding to the four red polygon regions shown in Fig. 2: the Mid-Atlantic, West Florida, Louisiana, and Texas coasts.

**Fig. 4:**
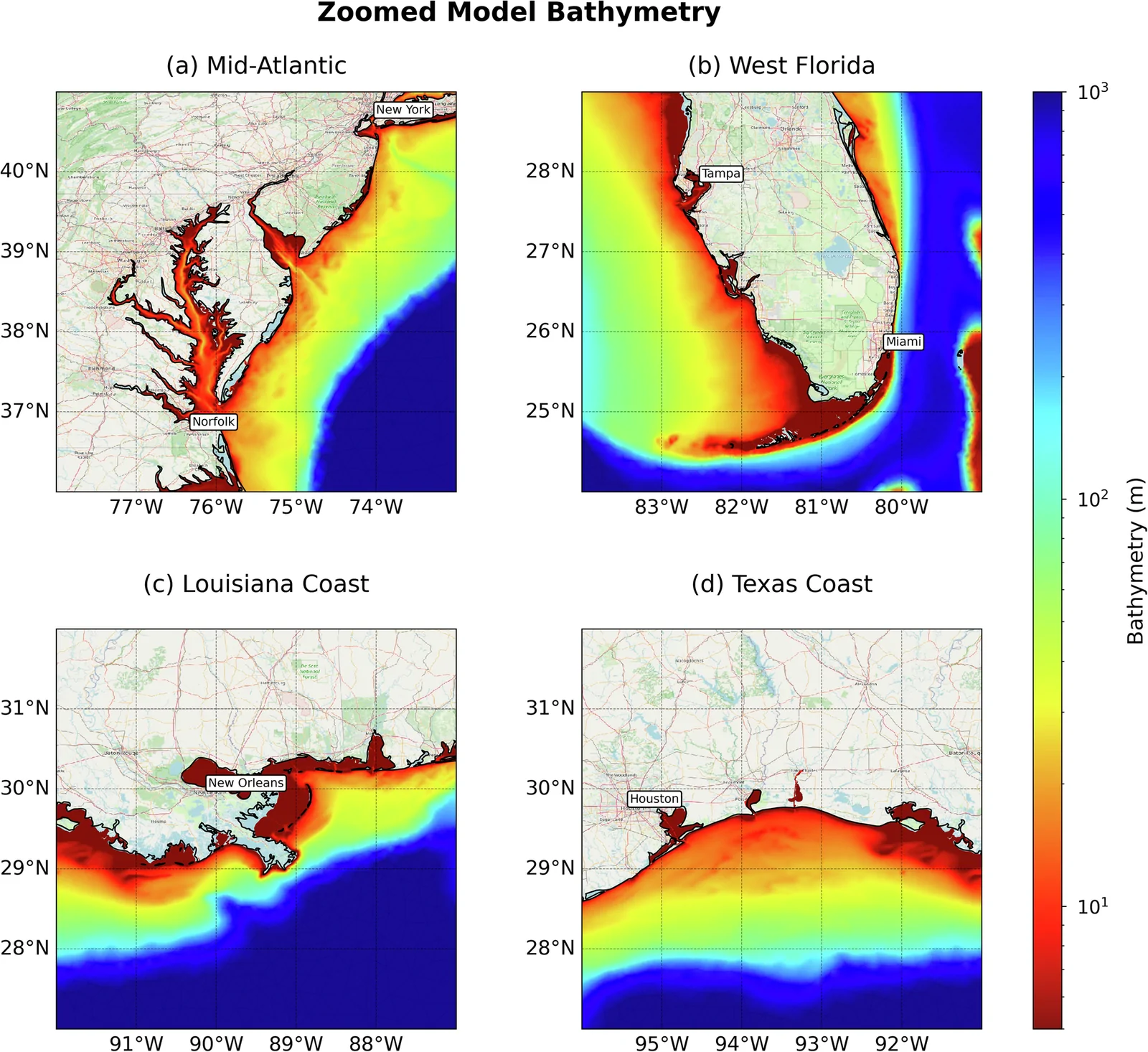
Inset views of the model grid bathymetry corresponding to the four red polygon regions shown in Fig. 2: the Mid-Atlantic, West Florida, Louisiana, and Texas coasts.

We developed our model grid to achieve a balance between resolution and computational efficiency for large-ensemble storm surge simulations, shown in Figs. 2 and 3. Our mesh comprises 337,263 nodes and 643,912 elements, significantly fewer than the 2.07 million nodes and 3.77 million elements used in EC2015. This reduction makes it feasible to conduct hundreds of hurricane simulations. Although coarser overall, we still achieve 200 m nearshore resolution in critical regions to capture the dominant tide, surge, and wave dynamics. Figs. 3 and 4 illustrate various coastal areas of the U.S. - specifically, the Mid-Atlantic, West Florida, Louisiana, and Texas - that are particularly vulnerable to frequent hurricane landfalls and the associated storm surges. Figure 3 displays the variation in model resolution, while Fig. 4 shows the bathymetry, highlighting the transition from deep water to shallow coastal areas. We generated the grid using the Surface-water Modeling System (SMS v13.0, Aquaveo LLC) with a paving density function that scales element size with the variations in bathymetry gradient, wavelength, and wave celerity. In contrast to Szpilka *et al*.^30^, we strictly followed the shoreline boundary and did not extend the mesh onto dry land or narrow channels/inlets. This approach avoided model instabilities due to wetting and drying for our extensive storm cases while substantially lowering the computational cost.

### Model Forcings: Wind field and Tidal boundary condition

**Fig. 5:**
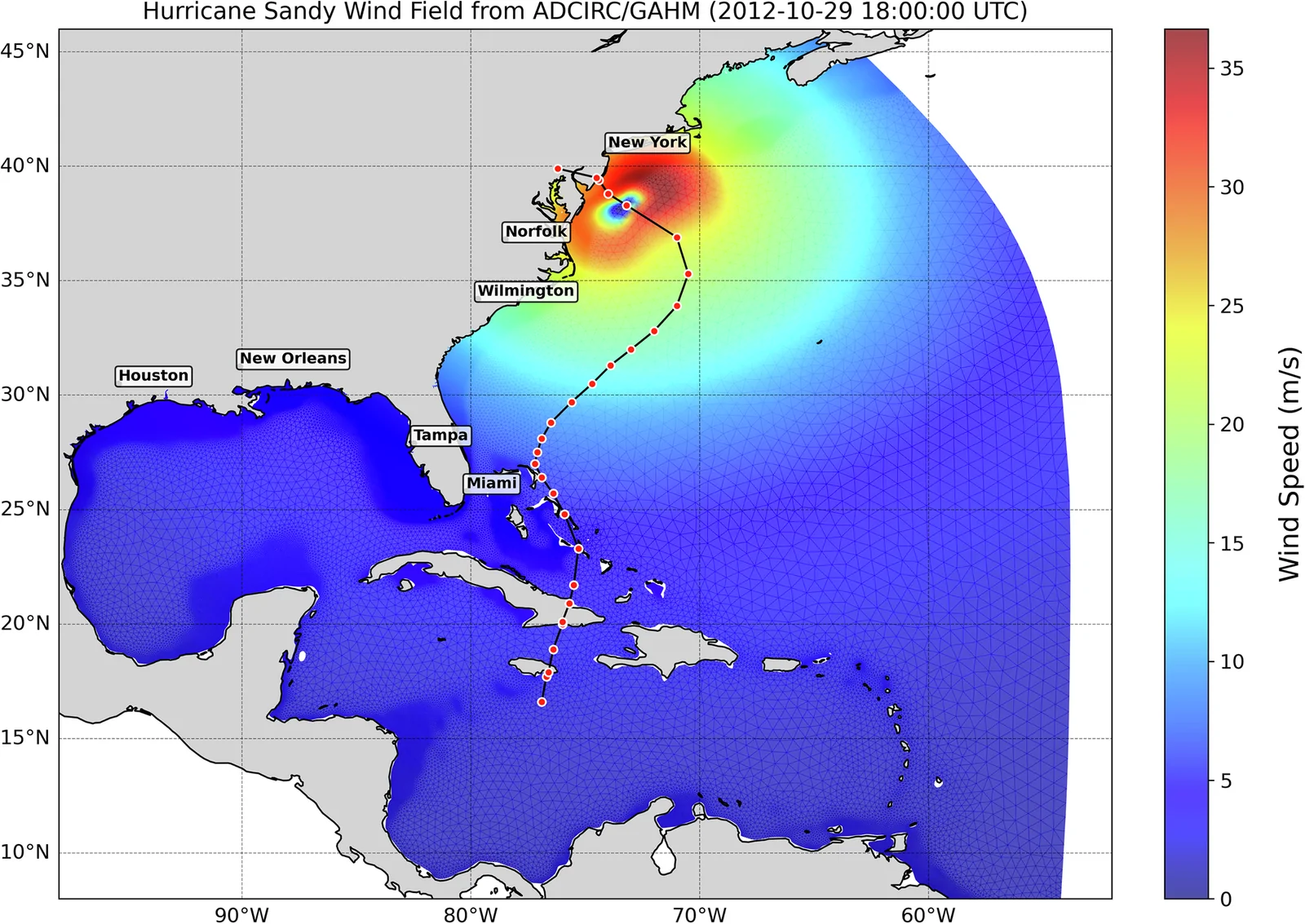
The instantaneous wind field generated by GAHM on the ADCIRC model grid shows the landfalling wind speed of Hurricane Sandy (2012) along with the hurricane track. The black line represents the track, and the red circles are storm positions at every six-hour intervals.

The post-storm NHC best-track records in ATCF format provide a  ~ 6-hourly time series of storm parameters, center position and motion, minimum central pressure, maximum sustained wind, and quadrant-specific radii of the 34/50/64-kt isotachs (NE/SE/SW/NW). We postprocess these tracks and provide them as fort.22 files to ADCIRC, where ADCIRC uses the Generalized Asymmetric Holland Model (GAHM, a parametric hurricane vortex model based on the classical Holland Model^32^), to construct the asymmetric hurricane wind field. We provided an example of ADCIRC and GAHM-generated instantaneous wind field in Fig. 5, which shows the landfalling wind speed of Hurricane Sandy (2012) along with the hurricane track. In coupled runs, ADCIRC computes water level and currents using the GAHM-generated 10-m winds and sea-level pressure, while SWAN is forced with the same winds to resolve wave radiation stress. The fort.22 file is generated using the ASWIP tool (distributed with the ADCIRC model code), which converts the NHC best-track ATCF record into the parametric vortex format required by NWS = 320. ADCIRC’s momentum equations include the atmospheric pressure gradient term ($-\frac{1}{\rho }\nabla {p}_{a}$), so the simulated water levels incorporate the inverse barometer effect; the dynamic (non-isostatic) ocean response to the translating pressure anomaly is captured through ADCIRC’s time-dependent shallow-water equations. The GAHM model is sensitive to fort.22 formatting; it requires strict adherence to ASWIP output (an ADCIRC tool for fort.22 generation). Our sequence is: (1) acquire the NHC best-track ATCF file; (2) select storms whose tracks make U.S. landfall (or approach the coastline closely enough to generate significant surge), retaining the complete best-track record from genesis to dissipation for each selected storm; (3) insert the valid time of each best-track entry in column 6 while preserving the original delimiters exactly (otherwise ASWIP will not parse); and (4) pass the modified ATCF to ASWIP, which writes the fort.22 for NWS=20/320 meteorological forcing option in ADCIRC. Minor edits such as reformatting in a spreadsheet, altering commas/spaces, or tweaking ASWIP’s output can cause runtime errors. Many pre-1981 ATCF records lack key fields such as central pressure and/or Rmax; these cases require additional assumptions before using them in GAHM.

Using the OceanMesh2D MATLAB toolkit^33^, we obtained open-boundary tidal harmonics from TPXO9 (OSU Global Tidal Solutions^34^; data available at https://www.tpxo.net/home) for the eight constituents (Q1, O1, P1, K1, N2, M2, S2, K2). Specifically, we extracted the nodewise complex amplitudes (amplitude/phase). OceanMesh2D interpolates constituent amplitudes and phases at boundary nodes and writes them in ADCIRC’s required format using the Makef15 routine, designating TPXO9 as the tidal database. The boundary specification is consistent across all hurricane cases, effectively capturing the dominant diurnal and semidiurnal tides at the model boundary. While the harmonic constants (amplitudes and phases) for the tidal constituents remain uniform at the open boundary, ADCIRC internally adjusts the boundary forcing and tidal potential for each event based on its specific historical start date (specified per-storm in the fort.15 control file), accounting for the nodal factor (*f*) and equilibrium argument (*V*_0_ + *u*) associated with the 18.6-year nodal cycle. The tidal potential forcing (NTIP = 1) is computed using the same storm-specific start date.

### Model Control Variables, Constants, and Simulation Costs

We used the parallel MPI-enabled ADCIRC+SWAN model (v56.0.4) to perform coupled simulations. In all hurricane simulations, ADCIRC was configured with NWS = 320 to read the ASWIP-generated fort.22 inputs. Under this option, ADCIRC applies the parametric asymmetric wind and pressure forcing and, when coupled with SWAN, exchanges water levels and radiation stresses at the prescribed coupling interval, ensuring consistent surge-wave interaction throughout the storm period.

The model configuration in fort.15 was generated automatically using OceanMesh2D. A sponge layer along open-ocean boundaries (sponge_generator_layer) is assigned to absorb the outgoing wave energy, with input files (fort.53001, fort.54001) created using OceanMesh2D’s Calc_Sponge and Make_f5354 utilities. Simulations used geographic coordinates (ICS = 22) with Coriolis (NCOR = 1) and tidal potential forcing (NTIP = 1). Bottom friction followed a uniform quadratic drag coefficient (CF = 0.0025) as we did not include the inundation over land or vegetated areas, and horizontal mixing was represented by a Smagorinsky coefficient (ELSM = 4.0). A minimum wetting depth of 0.1 m is used to reduce model instability, and a 4-s time step satisfied the Courant stability criterion. ADCIRC was configured with a semi-implicit time integration scheme (IM = 0, equivalent to ILump = 0; consistent GWCE mass matrix solver) with GWCE time-weighting parameters A00 = 0.0, B00 = 1.0, C00 = 0.0, evaluating the GWCE at the present time level^35^; further details are available at https://wiki.adcirc.org/IM. Coupling between ADCIRC and SWAN occurred every 1,200 s (20 min) (RSTIMINC). We chose the Powell *et al*.^36^ wind-drag formulation and capped the drag coefficient at 0.003, with a background pressure of 1013 mb. This configuration remained consistent across all 232 historical hurricane simulations. A representative fort.15 file for Hurricane Sandy (2012) is provided in our dataset repository.

The SWAN model is configured to operate in a non-stationary mode using spherical coordinates consistent with the ADCIRC mesh. We assigned 36 directional bins and 40 frequency bins (0.031-1.42 Hz), resolving both swell and locally generated wind waves. Wave growth and dissipation followed the Komen *et al*.^37^ source-term formulation (GEN3 KOMEN AGROW), with white-capping defined by WCAP KOMEN. Depth-induced breaking (BREAKING) and bottom friction (FRICTION MADSEN KN = 0.05 m) accounted for nearshore energy loss.

The ADCIRC+SWAN model was compiled and executed at the Department of Energy’s (DOE) Perlmutter supercomputer, under the National Energy Research Scientific Computing Center (NERSC). In our Perlmutter setup (1 node, 64 MPI ranks, 2 OpenMP threads per rank; 128 cores total), a 6-day coupled ADCIRC+SWAN hindcast completed in approximately 1.0 wall-clock hour (throughput  ≈ 6 model days per wall-hour), corresponding to about 128 core-hours per hurricane case.

## Data Records

**Fig. 6:**
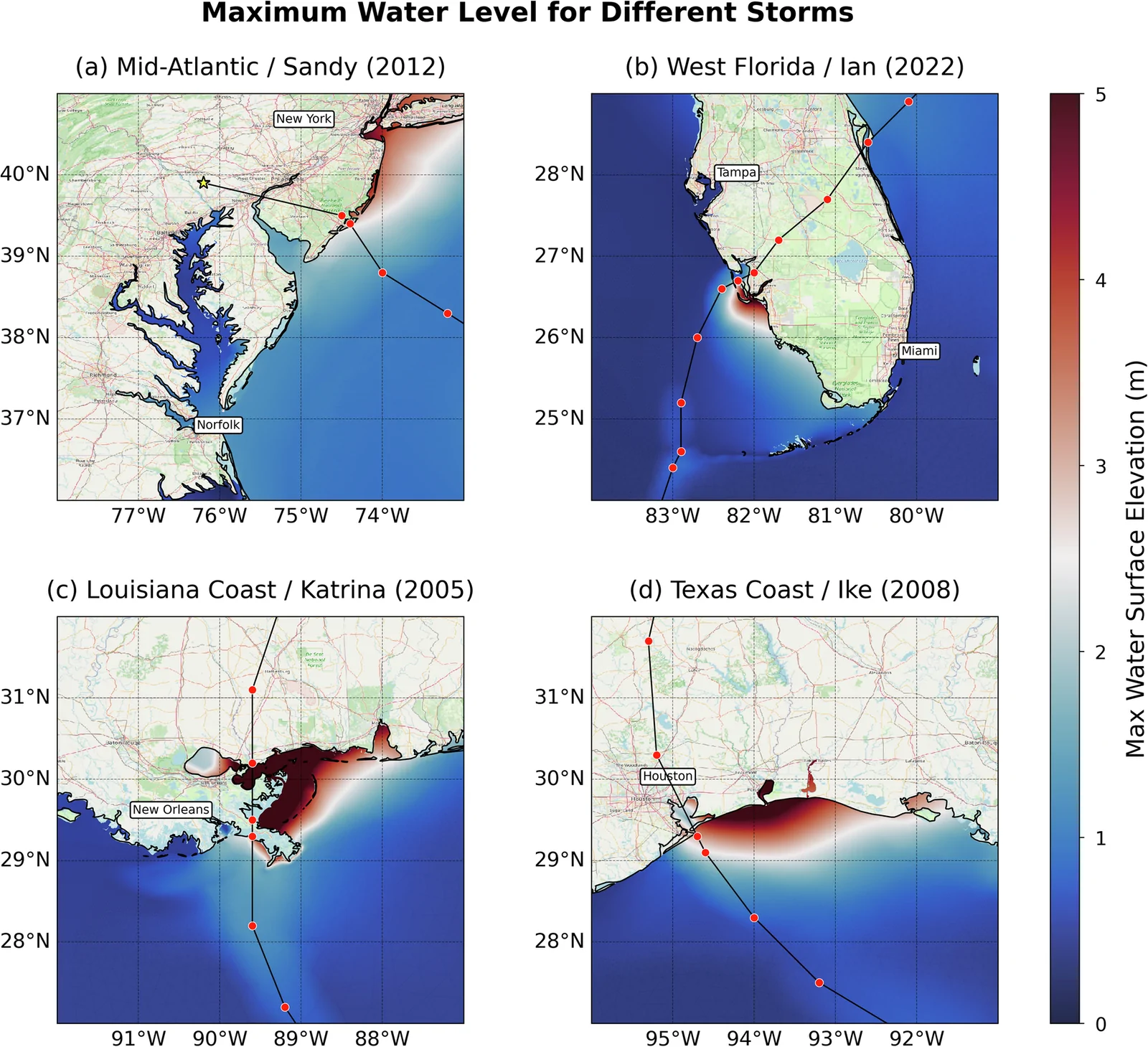
Maximum water surface elevation (meters) from coupled ADCIRC+SWAN hindcasts for four U.S. coastal regions and storms: (**a**) Mid-Atlantic — Hurricane Sandy (2012); (**b**) West Florida — Hurricane Ian (2022); (**c**) Louisiana — Hurricane Katrina (2005); (**d**) Texas — Hurricane Ike (2008). Each panel shows the spatial maximum over the hurricane window, highlighting regional differences in surge magnitude driven by storm characteristics and coastal setup. The color scale is consistent across all panels.

**Fig. 7:**
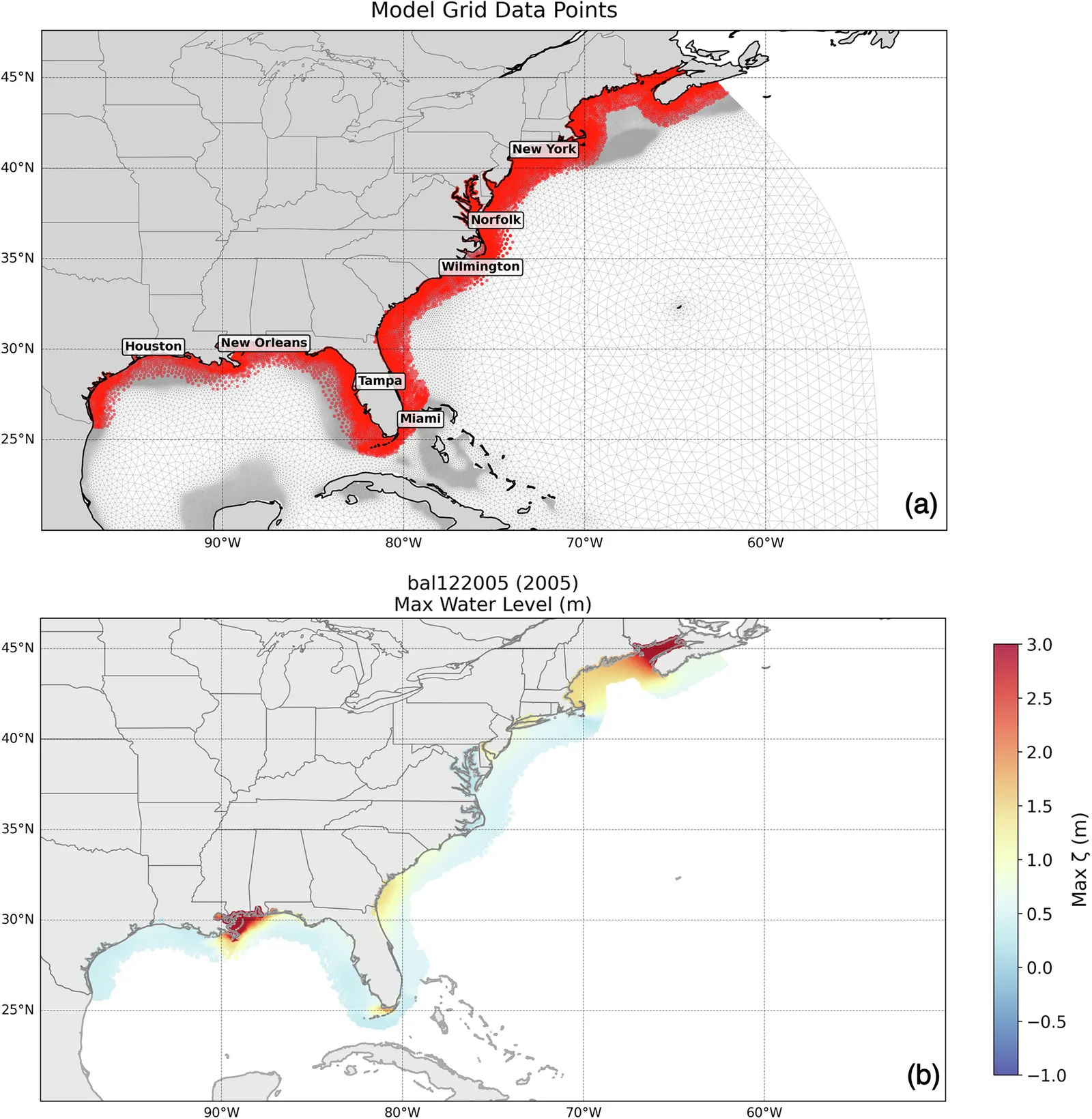
(**a**) Locations of model data extraction used to build the storm surge-wave database, superimposed on the unstructured ADCIRC+SWAN mesh. Gray markers show all mesh nodes; red markers indicate the subset of nodes where the time series of water surface and wave properties were sampled and written to the NetCDF files. At these points, users can access the variables: water surface elevation, significant wave height, and peak wave period, for each hurricane case; (**b**) Maximum water level during Hurricane Katrina (2005), [ATCF best track storm name: bal122005] at the data extraction locations shown in panel (**a**). Users can generate this plot using the script: extract_storm_spatial_map.py, provided in the Zenodo repository.

The data sets are available in a Zenodo repository, https://doi.org/10.5281/zenodo.17905131^38^, where we organised them by hurricane tracks (NHC best-track names). Decade archives (for example, AdcSwan_StormSurge_Data_1981_1990.nc) collect extractions across multiple events. Each decade file is CF-compliant (netCDF-4, compressed) and contains node coordinates (longitude(node), latitude(node)), an index of storm directories (subdir_names(name_strlen,subdir)), and three-dimensional model output variables with dimensions (node, time, subdir), such as water-surface elevation *ζ* (m), significant wave height swan_HS (m), and peak wave period swan_TPS (s). Time is provided in an authoritative character array time_str(time_strlen,time,subdir) containing ISO-8601 timestamps (YYYY-MM-DDTHH:MM:SS) for each storm timestep. Node longitude/latitude and variable attributes (long_name, units, standard_name) are included. Table 1 summarises the principal variables and their units. In Fig. 6, we show the maximum water surface elevation at four U.S. vulnerable regions, Mid-Atlantic, West Florida, Louisiana, and Texas coast, based on the model results for different hurricanes that impacted those regions. We also show the model nodes or grid points used for data extraction and preparing the database in Fig. 7a. The red points overlayed on the model grid nodes, shown in gray color, represent the points from which the users can extract and use the model output variables saved in the netcdf files. Figure 7b shows the maximum water level in the same grid nodes during Hurricane Katrina (2005).

**Table 1:** Summary of ADCIRC+SWAN NetCDF storm-surge datasets.

NetCDF file	Variables (size and dimensions)
AdcSwan_StormSurge_Data_1981_1990.nc	longitude: 12536 × 1 (node);
	latitude: 12536 × 1 (node);
	time_str: 19 × variable × 37 (time_strlen, time, subdir; ISO-8601);
	subdir_names: 9 × 37 (name_strlen, subdir);
	swan_HS: 12536 × variable × 37 (node, time, subdir);
	swan_TPS: 12536 × variable × 37 (node, time, subdir);
	zeta: 12536 × variable × 37 (node, time, subdir)
AdcSwan_StormSurge_Data_1991_2000.nc	time_str: 19 × variable × 44 (time_strlen, time, subdir; ISO-8601);
	subdir_names: 9 × 44 (name_strlen, subdir);
	swan_HS: 12536 × variable × 44 (node, time, subdir);
	swan_TPS: 12536 × variable × 44 (node, time, subdir);
	zeta: 12536 × variable × 44 (node, time, subdir)
AdcSwan_StormSurge_Data_2001_2010.nc	time_str: 19 × variable × 72 (time_strlen, time, subdir; ISO-8601);
	subdir_names: 9 × 72 (name_strlen, subdir);
	swan_HS: 12536 × variable × 72 (node, time, subdir);
	swan_TPS: 12536 × variable × 72 (node, time, subdir);
	zeta: 12536 × variable × 72 (node, time, subdir)
AdcSwan_StormSurge_Data_2011_2021.nc	time_str: 19 × variable × 75 (time_strlen, time, subdir; ISO-8601);
	subdir_names: 9 × 75 (name_strlen, subdir);
	swan_HS: 12536 × variable × 75 (node, time, subdir);
	swan_TPS: 12536 × variable × 75 (node, time, subdir);
	zeta: 12536 × variable × 75 (node, time, subdir)

The longitude and latitude variables are included in all files but listed here only once for brevity. The subdir dimension corresponds to individual storm names derived from the NHC best-track database, and the time dimension length varies by storm duration. Each simulation provides hourly output and typically spans 5–10 days depending on the hurricane lifecycle. The time_str variable contains ISO-8601 formatted datetime strings (YYYY-MM-DDTHH:MM:SS) derived from the simulation start time in the NHC best-track file (fort.22) and ADCIRC numeric time (seconds)

## Technical Validation

**Fig. 8:**
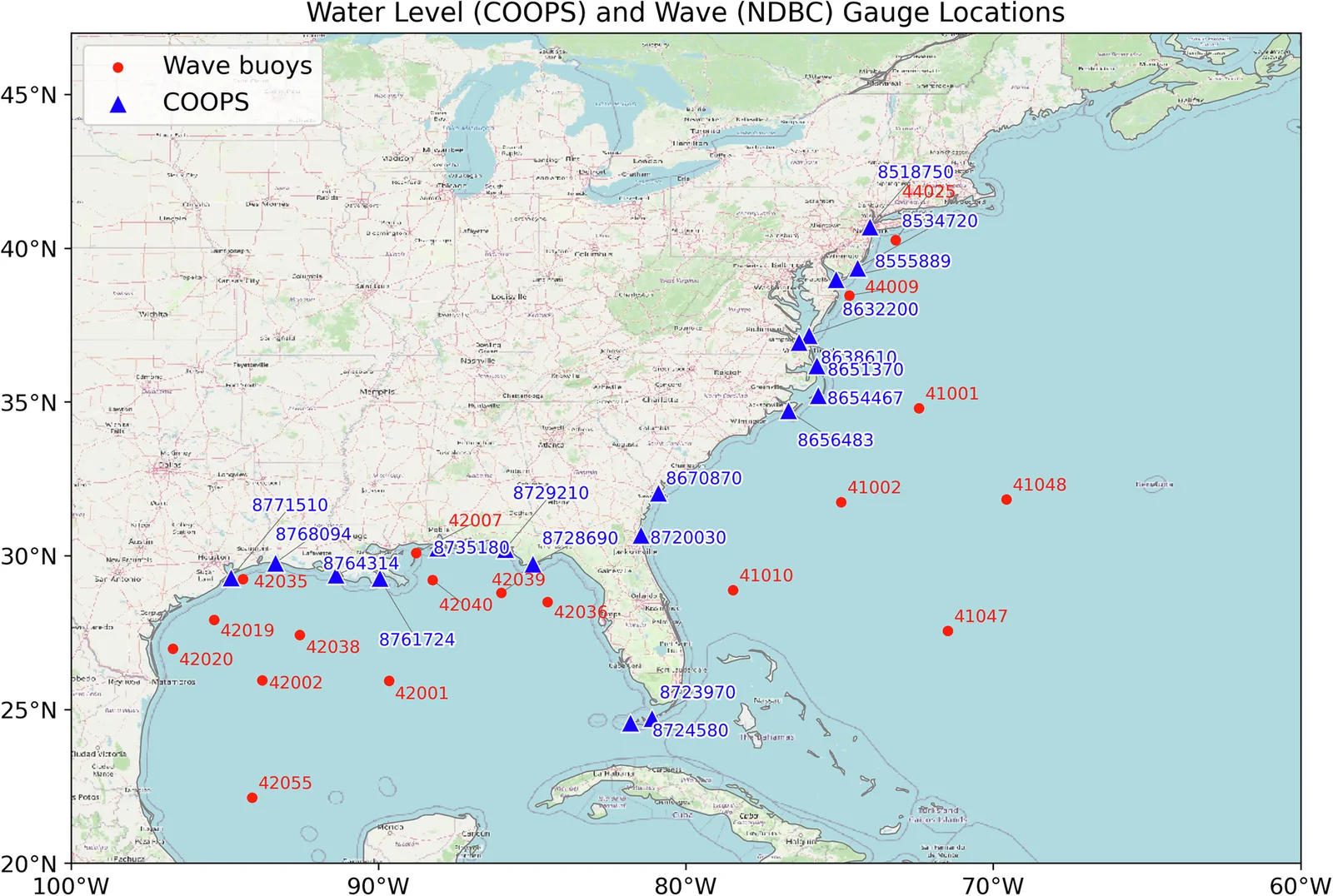
NOAA and NDBC field gauge locations used to compare four key variables—water surface elevation at 23 NOAA tide gauge locations, significant wave height (Hs) and peak wave period (Tp) at 23 NDBC wave buoy stations, and 10-m elevation wind speed at four meteorological stations—for different hurricanes from 2003 to 2022.

We examined the model performance by doing a comprehensive validation against observational data for ten representative hurricanes spanning 2003-2022: Isabel (2003), Katrina (2005), Ike (2008), Irene (2011), Sandy (2012), Matthew (2016), Michael (2018), Laura (2020), Ida (2021), and Ian (2022). We selected these specific events to represent diverse storm characteristics and geographic regions across the U.S. coastline. Also, continuous water level data are available from multiple NOAA tide gauge locations during these events. We compared four key variables: water surface elevation (or water level) at 23 NOAA tide gauge locations, significant wave height (Hs) and peak wave period (Tp) at 23 NDBC wave buoy stations, and 10-m elevation wind speed at four meteorological stations, shown in Fig. 8. We extracted the model time series for each validation case at the precise geographic coordinates of observation stations and compared them with observations in a consistent way. We also performed robust quality control to exclude physically unrealistic values while preserving realistic peak values during storm conditions. We computed four standard performance metrics for all comparisons: Pearson correlation coefficient (R), normalized bias, Willmott skill score, and peak difference (absolute and percentage). They provide a comprehensive assessment of temporal correlation, systematic bias, overall predictive skill, and accuracy in our comparisons. When we compared the model-provided water level relative to MZ with field measurements (relative to local Mean Sea Level), we observed a systematic offset that varied by location. This offset shows that the MZ datum differs slightly from the local MSL at each gauge site. We quantified these differences and observed that the required adjustment ranged from approximately 0 to 0.4 m across the study area. Before comparing the model results with observations, we applied location-specific vertical corrections to the model output. By making these adjustments, we kept the model and the observed water levels expressed relative to the local MSL datum, allowing for consistent comparisons across regions.

### Water Level

**Fig. 9:**
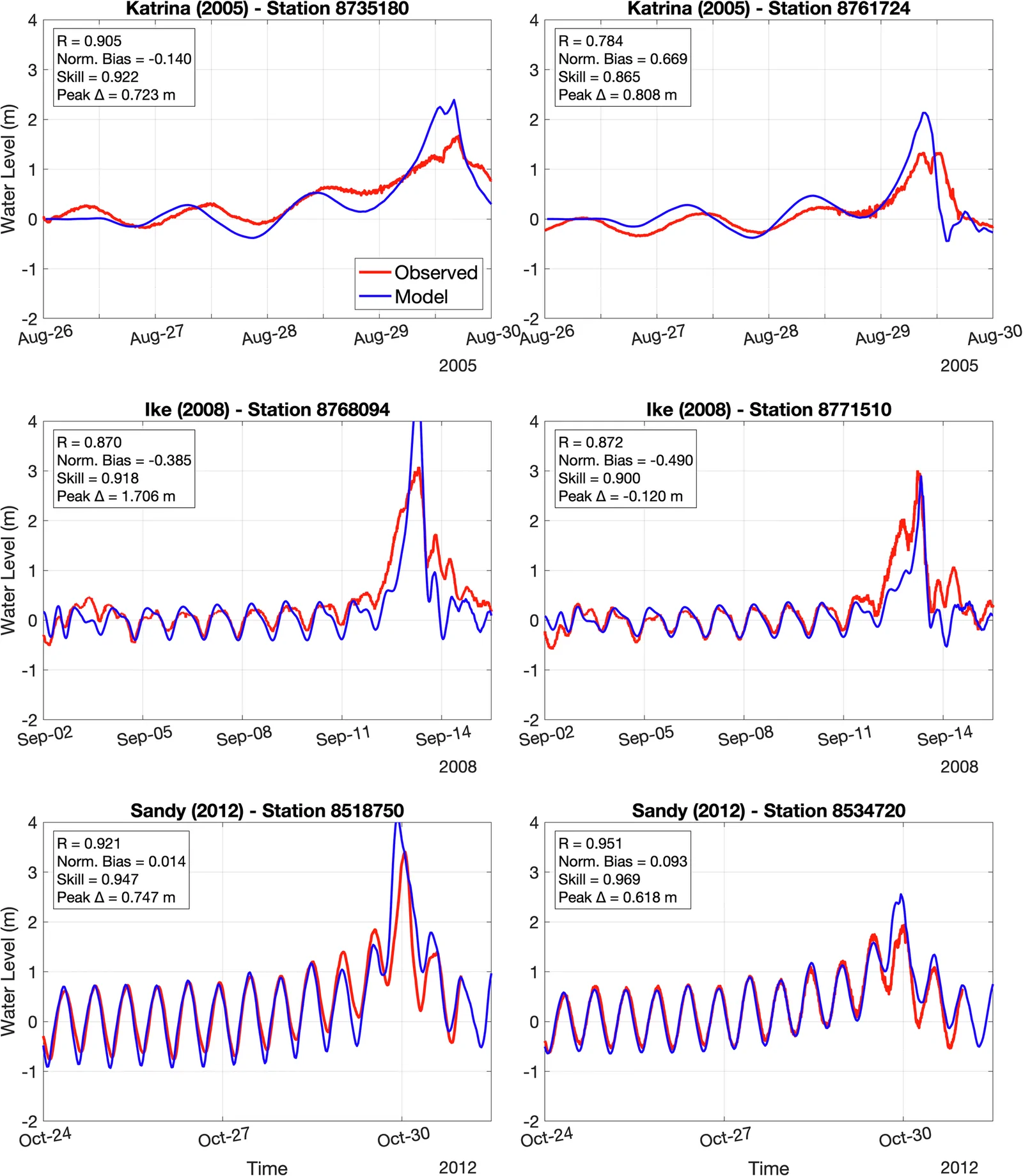
Time-series comparison of model (blue) and observed (red) water surface elevation for three hurricane events, Hurricane Katrina (2005), Hurricane Ike (2008), and Hurricane Sandy (2012), at selected NOAA tide gauge stations.

The predictions of water surface elevation generally compare well, but we also observe significant geographic and storm-dependent variations across different regions (see Table 2). In Gulf Coast locations impacted by Hurricanes Katrina and Ike, we observed high correlation coefficients (R > 0.85), with peak water-level differences typically staying within 2 meters and normalized biases generally around 15% or better. However, there are some outliers: Hurricane Laura (2020) at gauge 8768094 displayed the largest normalized bias (12.96%), while Hurricane Ida (2021) at gauge 8761724 exhibited a severe negative bias (−45.11%), resulting in peak differences exceeding 1.4 meters (148%). In the Mid-Atlantic region, stations that recorded water levels during Hurricanes Irene and Sandy demonstrated moderate prediction skill (R = 0.71–0.95) with smaller biases. Figure 9 compares water level time series for three significant events: Hurricane Katrina (2005), Hurricane Ike (2008), and Hurricane Sandy (2012), which made landfall in different regions. We selected two gauge locations near the coast and show the comparisons side by side. The model predicts tidal variations well. However, differences between the model and observed peak water levels vary by location and event, as the model tends to overestimate peak water levels in most cases (consistent with comparable 2D barotropic storm surge studies over the eastern continental shelf; e.g., Zhang and Sheng^39^). The physical drivers of this overestimation—including the absence of a floodplain and the GAHM peak wind bias—are discussed in the “Known Limitations” section.

**Table 2:** Water surface elevation validation results for ten hurricane events and 23 tide gauges.

Storm	Gauge ID	R	Norm. Bias	Skill	Peak Diff. (m)	Peak Diff. (%)
Isabel (2003)	8632200	0.87	−8.92	0.88	1.25	109.2
Isabel (2003)	8638610	0.85	1.55	0.87	2.02	122.2
Katrina (2005)	8735180	0.90	−0.14	0.92	0.72	64.9
Katrina (2005)	8761724	0.78	0.65	0.87	0.81	98.9
Ike (2008)	8768094	0.86	−0.36	0.91	1.71	59.7
Ike (2008)	8771510	0.87	−0.47	0.90	−0.12	40.4
Irene (2011)	8555889	0.94	−0.16	0.97	0.09	39.3
Irene (2011)	8651370	0.94	0.41	0.97	0.11	56.0
Irene (2011)	8654467	0.71	2.52	0.81	0.14	111.0
Irene (2011)	8656483	0.93	−0.41	0.94	0.02	48.4
Sandy (2012)	8518750	0.92	0.01	0.95	0.75	61.2
Sandy (2012)	8534720	0.95	0.09	0.97	0.62	54.5
Matthew (2016)	8670870	0.87	−0.82	0.92	0.35	63.8
Matthew (2016)	8720030	0.82	−0.93	0.89	−0.26	54.7
Michael (2018)	8728690	0.88	0.32	0.94	0.65	36.2
Michael (2018)	8729210	0.72	−0.13	0.79	−0.56	91.8
Laura (2020)	8764314	0.77	1.08	0.87	0.42	56.2
Laura (2020)	8768094	0.87	12.96	0.92	0.39	27.6
Ida (2021)	8741533	0.89	−0.18	0.93	−0.12	46.7
Ida (2021)	8747437	0.93	−0.23	0.94	−0.47	42.3
Ida (2021)	8760922	0.87	−0.24	0.93	0.22	42.6
Ida (2021)	8761724	0.86	−5.11	0.85	1.43	148.7
Ian (2022)	8724580	0.91	−1.15	0.93	−0.30	45.2

### Significant Wave Height

After the water level comparison, we evaluated significant wave height errors across 23 NDBC buoy stations (Fig. 8). We observed that the model predictions at offshore sites consistently show larger errors than at nearshore sites. The statistics in Table 3 reveal consistent patterns across storms: offshore locations exhibit larger Hs errors. During Hurricane Katrina, the model prediction at offshore Gulf buoys 42001 and 42002 performed poorly. At 42001, we measured a high correlation (R = 0.93) but found a large positive normalized bias (56.5%) and a 7.67 m peak difference (118% relative error), while nearshore buoy 42019 maintained R = 0.95 with only 14% bias. At 42002, the model yielded a low correlation (R = 0.366) and a large normalized bias (90.8%), with a peak error of 140.8%. In contrast, nearshore Katrina buoys (42019, 42036-42040) performed substantially better (R = 0.76-0.97, normalized biases 5%–29%, peak errors 26%–56%). This higher offshore bias persisted across other events: at Ike’s offshore buoy 42001, we recorded a 46.5% normalized bias with R = 0.69, and at Sandy’s offshore buoy 44025, the bias reached 44.4%. We observed that deep-water sites routinely exceed 40% normalized bias, whereas coastal gauges typically have bias below 30%. Several stations display high correlations but show significant amplitude errors, indicating that while the model accurately captures the timing of wave evolution, it consistently overestimates the magnitudes at these locations.

**Table 3:** Significant wave height validation results for three hurricane events and 23 wave buoy stations.

Storm	Buoy ID	R	Norm. Bias	Skill	Peak Diff. (m)	Peak Diff. (%)
Katrina	42001	0.93	0.57	0.55	7.67	118.3
Katrina	42002	0.37	0.91	0.33	3.79	140.9
Katrina	42019	0.95	0.14	0.92	0.55	26.2
Katrina	42020	0.79	0.70	0.63	1.89	88.8
Katrina	42036	0.80	0.05	0.76	0.14	50.9
Katrina	42038	0.96	0.21	0.82	3.16	56.0
Katrina	42039	0.76	0.08	0.76	0.29	34.1
Katrina	42040	0.97	0.28	0.87	3.85	35.9
Katrina	42055	0.84	0.51	0.60	1.35	68.0
Ike	42001	0.70	0.47	0.48	5.39	69.2
Ike	42002	0.97	0.56	0.48	3.76	78.3
Ike	42007	0.45	-0.04	0.66	-0.24	100.0
Ike	42019	0.89	0.41	0.69	2.16	58.9
Ike	42020	0.92	0.19	0.90	0.62	39.4
Ike	42035	0.82	0.03	0.90	0.34	33.5
Ike	42040	0.81	0.05	0.88	0.13	100.0
Sandy	41001	0.67	0.28	0.53	2.86	45.9
Sandy	41002	0.88	0.50	0.48	4.35	64.4
Sandy	41010	0.80	0.13	0.65	2.06	37.5
Sandy	41047	0.83	0.04	0.89	-0.75	37.2
Sandy	41048	0.67	0.26	0.63	1.06	41.4
Sandy	44009	0.78	0.19	0.76	2.42	58.5
Sandy	44025	0.80	0.44	0.60	5.24	77.2

**Fig. 10:**
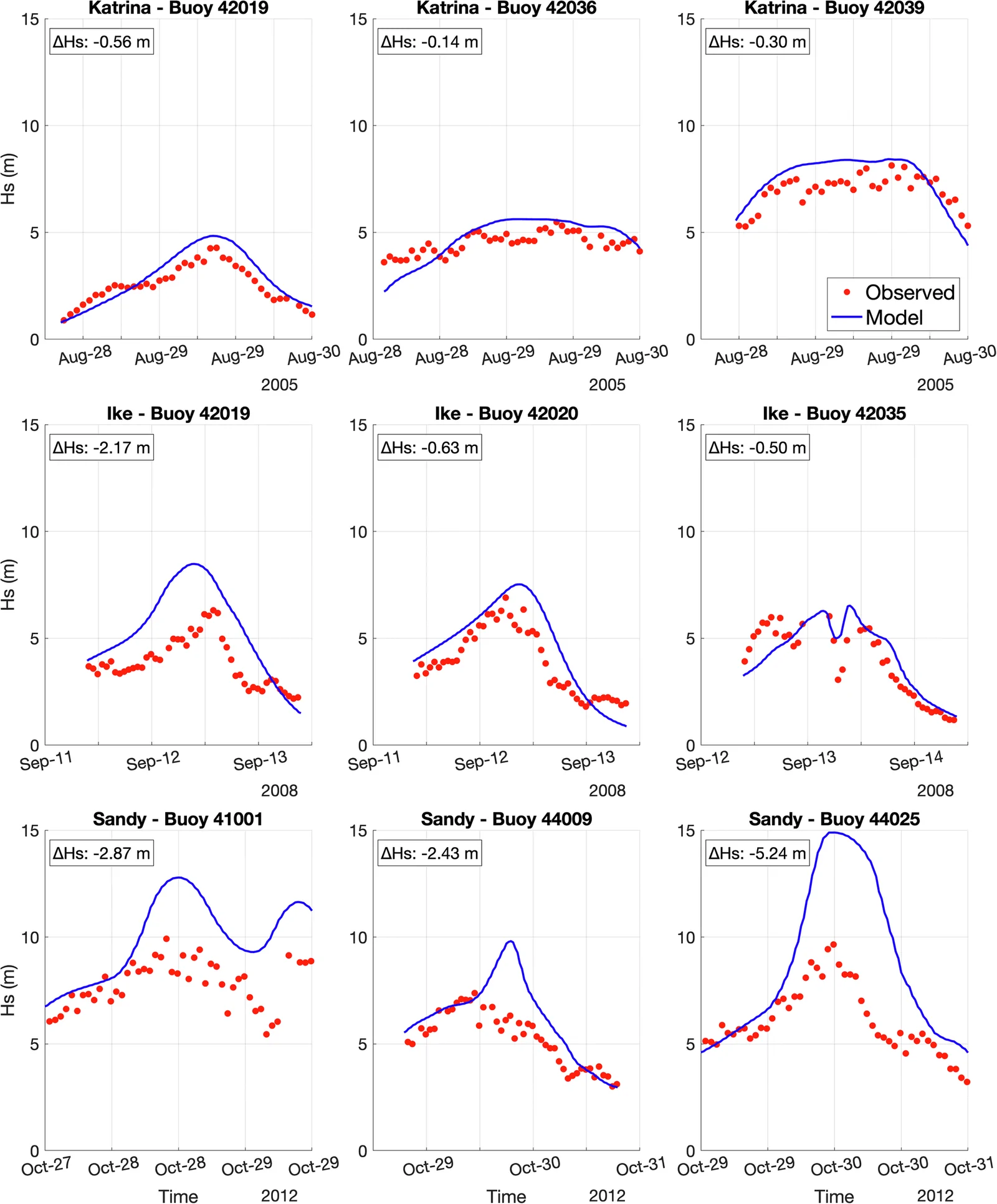
Time-series comparison of model (blue) and observed (red) significant wave height for three hurricane events, Hurricane Katrina (2005), Hurricane Ike (2008), and Hurricane Sandy (2012), at selected NOAA NDBC wave buoys.

The model predicts larger significant wave heights than NDBC observations in almost all validation cases, and this systematic positive bias is pronounced at offshore locations. The trend of overestimating wave heights is consistent across various storm types and geographic regions, suggesting a general tendency toward overprediction. However, we note that the offshore biases are especially pronounced in the Gulf of Mexico. Fan *et al*.^40^ stated that mesoscale ocean features such as the Loop Current can significantly modify the wave field through wave-current interactions. Although ADCIRC+SWAN simulations are coupled, its 2D barotropic formulation and our short simulation periods limit the development of the Loop Current and its associated mesoscale circulation features. This limitation may partly account for the larger offshore biases observed in the Gulf. Lin *et al*.^41^ further demonstrated, using a fully 3D coupled ROMS–SWAN system, that explicitly resolving three-dimensional wave–current interactions significantly reduces the positive wave height bias relative to 2D-coupled configurations; the same mechanism—depth-averaged rather than surface-layer currents being passed to SWAN—contributes to the systematic overestimation documented here. We presented a comparison plot, Fig. 10, illustrating the time series of significant wave heights for three hurricanes: Hurricane Katrina (2005), Hurricane Ike (2008), and Hurricane Sandy (2012). These are the same hurricanes we analyzed for the water level comparison in Fig. 9. The results indicate that the peak wave height for Hurricane Sandy (2012) is significantly higher, while the model performs better for Hurricanes Katrina (2005) and Ike (2008).

The systematic bias at offshore locations is consistent with previous studies and can be attributed to several factors. First, the wind drag parameterization plays a critical role: Fan *et al*.^40^ demonstrated that wave models, such as WAVEWATCH III, tend to overestimate significant wave height and dominant wavelength under extreme tropical cyclone conditions from inadequate drag parameterization. Huang *et al*.^42^ further quantified this effect for Hurricane Ike, showing that SWAN’s default wind input parameterization overestimates maximum significant wave heights by approximately 2 meters in deep Gulf of Mexico waters, and this overprediction can be remedied by adjusting the maximum surface drag coefficient. Second, the choice of wind forcing significantly influences wave predictions. Bennett and Mulligan^43^ compared parametric wind models (Holland H80 and GAHM) against a regional atmospheric model for Hurricane Sandy and found that GAHM tends to over-predict significant wave height, while atmospheric models with data assimilation yield better agreement with observations. Third, the source-term parameterization in SWAN affects model accuracy in a storm-dependent manner. Loveland *et al*.^44^ quantified these effects for Hurricanes Ike (2008) and Ida (2021), finding that upgrading from first- or second-generation source terms to the ST6 third-generation package resulted in approximately 20% improvement in significant wave height accuracy during Hurricane Ike. Arab *et al*.^45^ examined wave sensitivity in the northeastern Gulf of Mexico and noted that the Janssen generation scheme tends to overestimate wave heights during Hurricane Ida (2021), while also emphasizing that differences between wind products are crucial for realistic hurricane-induced wave estimations and can be attributed to atmospheric model configuration and physics. Hope *et al*.^46^ validated the coupled SWAN+ADCIRC model during Hurricane Ike (2008) and confirmed that SWAN performs well in nearshore areas, consistent with our findings that also show substantially lower biases than offshore buoys.

### Peak Wave Period

Wave period predictions show the poorest overall performance, with substantial errors across multiple storms and locations. Table 4 shows distinctly different error patterns between Gulf and Atlantic storms. Hurricane Sandy (2012) produced mixed results: buoy 41001 shows a negative correlation, while other stations (41010, 41047, 41048) performed much better, with peak period differences of only 53–61%, and some achieved high skill scores (e.g., 41047: R = 0.86, skill = 0.85). In contrast, storms in the Gulf Coast showed systematic overprediction. We measured large peak period errors during Katrina (180–205% of observed values) with correlations ranging from 0.24 to 0.91, and Ike (2008) showed similarly high percentage errors (170–200% bias) despite better correlations. Many stations also demonstrated a correlation-skill disconnect, where moderate temporal correlations (R = 0.6-0.8) coincided with poor skill scores (0.3-0.5). The magnitude of period errors consistently exceeded wave height biases, with most Gulf Coast stations showing percentage errors exceeding 160%.

**Table 4:** Peak wave period validation results for three hurricane events and 23 wave buoy stations.

Storm	Buoy ID	R	Norm. Bias	Skill	Peak Diff. (s)	Peak Diff. (%)
Katrina	42001	0.40	0.23	0.48	3.38	197.6
Katrina	42002	0.24	0.56	0.40	3.25	182.9
Katrina	42019	0.57	0.37	0.48	3.18	166.1
Katrina	42020	0.75	0.24	0.53	3.33	168.0
Katrina	42036	0.28	0.24	0.31	3.48	167.1
Katrina	42038	0.91	0.27	0.72	4.28	184.5
Katrina	42039	0.81	0.28	0.30	2.84	194.8
Katrina	42040	0.59	0.33	0.39	4.73	195.0
Katrina	42055	0.45	0.36	0.37	3.15	205.2
Ike	42001	0.68	0.15	0.66	0.59	172.0
Ike	42002	0.83	0.27	0.69	1.67	173.1
Ike	42007	0.80	0.21	0.59	0.05	176.1
Ike	42019	0.76	0.32	0.62	4.11	198.0
Ike	42020	0.86	0.19	0.84	1.82	174.9
Ike	42035	0.88	0.20	0.85	2.27	182.0
Ike	42040	0.75	0.15	0.56	0.77	181.3
Sandy	41001	-0.41	0.25	0.28	1.94	91.2
Sandy	41002	0.38	0.25	0.33	2.45	92.6
Sandy	41010	0.67	0.08	0.65	-0.09	60.6
Sandy	41047	0.86	0.08	0.85	-0.12	56.3
Sandy	41048	0.37	0.18	0.40	1.36	53.0
Sandy	44009	0.51	0.35	0.55	1.36	187.0
Sandy	44025	0.43	0.32	0.36	5.05	170.7

The tendency for SWAN to overestimate peak wave period under hurricane conditions has been documented in previous studies. Montoya *et al*.^47^ performed a comparison of SWAN and WAVEWATCH III using observed data from Hurricane Katrina (2005) and found that SWAN consistently overestimates the peak wave period for almost all analyzed NOAA buoys during hurricane conditions. Hope *et al*.^46^ similarly indicated that the SWAN model slightly overestimates peak wave period at specific gauges during Hurricane Ike (2008), suggesting a persistent bias in period prediction. This bias could be related to the whitecapping dissipation formulation. Bhaskaran *et al*.^48^ demonstrated that employing a modified whitecapping dissipation formulation can improve wave period prediction compared to the default SWAN model. Joyce *et al*.^49^ reported that the ADCIRC+SWAN model, when forced by the Ocean Weather Inc. (OWI) data-assimilated wind product, yielded the lowest mean absolute error and root mean square error for mean wave period.

We note that the observed peak wave period data contained significant noise and temporal gaps, mainly during extreme conditions, which complicated direct model-observation comparisons and may have also contributed to some of the large biases and inconsistencies. These results show that wave period prediction faces basin-specific challenges: hurricanes along the Gulf Coast lead to systematic overprediction, while Atlantic storms exhibit greater variability in performance depending on storm characteristics and location.

### GAHM-generated Wind Speed

**Fig. 11:**
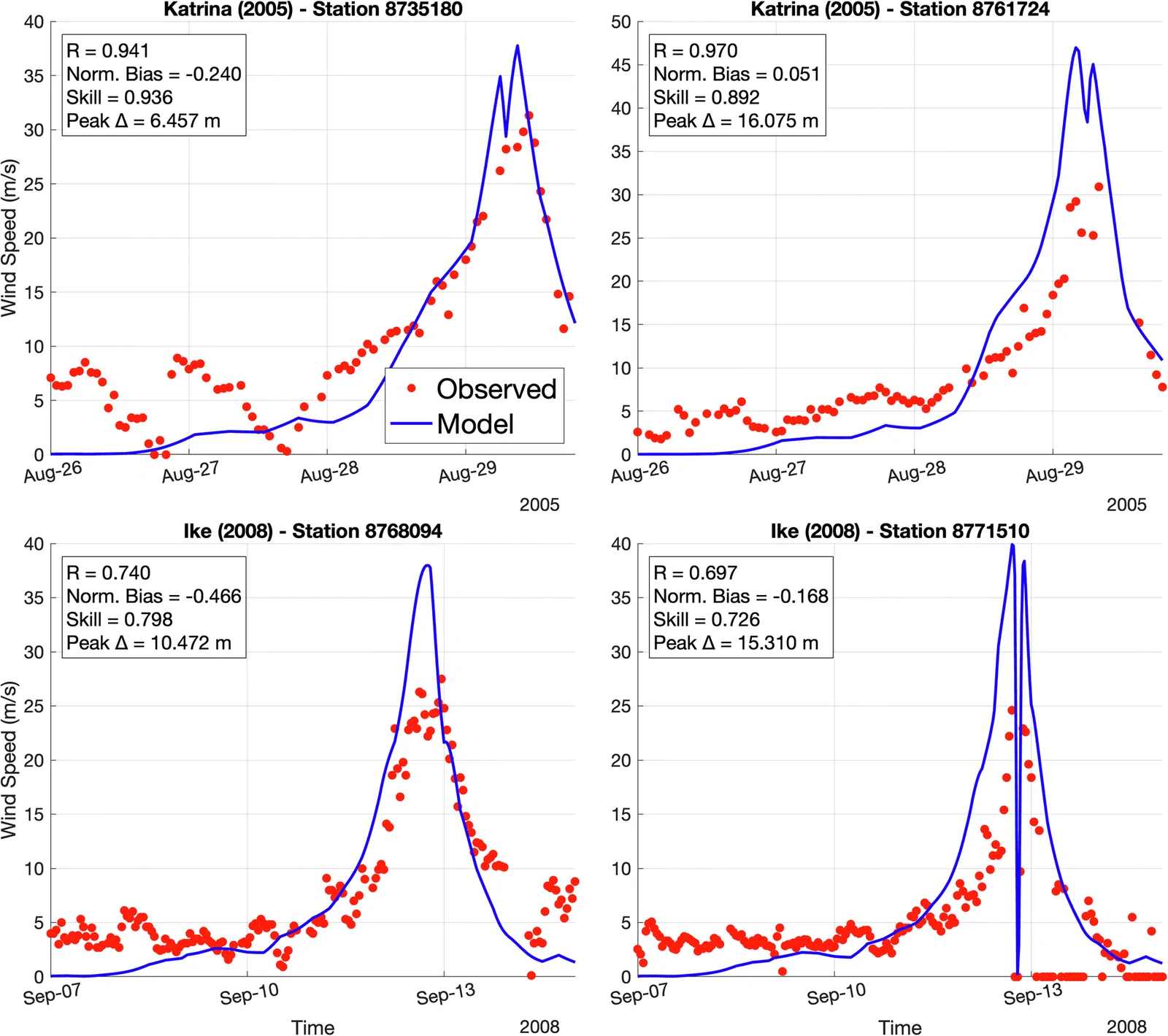
Time-series comparison of GAHM model (blue) and observed (red) surface wind speed (10-m elevation) for two hurricane events, Hurricane Katrina (2005) and Hurricane Ike (2008), at selected NOAA gauges.

Finally, we analyzed surface wind (10-m elevation) at four NOAA stations to understand how it contributes to errors observed in water level and wave data comparisons, shown in Fig. 11. This analysis shows systematic patterns that clarify the biases in wave predictions (Table 5). We observed generally strong correlations (R =0.69-0.97) but overall negative normalized biases (− 46.6% *to *5.1%) across most stations. While the GAHM model underrepresents wind speeds before and after hurricane passage (contributing to the negative overall bias), peak wind speed differences show consistent overestimation during the landfalls. All four stations show positive peak differences ranging from 6.5 to 16.1 m/s, indicating that the model systematically overestimates maximum wind speeds despite the negative time-averaged bias. For example, during Katrina, station 8735180, exhibits − 24% normalized bias but a 6.5 m/s peak overestimate, while during Ike, station 8768094, shows − 47% overall bias yet 10.5 m/s peak overestimation. This overestimation of peak intensity helps explain the systematic wave height and period overprediction we observed by the model. Previous studies Bennett and Mulligan^43^ have shown that parametric hurricane wind models like GAHM struggle with accurate temporal wind field representation throughout the storm evolution, particularly in capturing the appropriate intensity gradients. Future works would benefit from higher-resolution wind datasets or hybrid approaches that blend parametric models with reanalysis products (e.g., ERA5+GAHM) or commercial wind datasets (e.g., OWI^49^) to achieve more accurate wave predictions across the storm evolution.

**Table 5:** Wind speed validation results for two hurricane events and four meteorological stations.

Storm	Station ID	R	Norm. Bias	Skill	Peak Diff. (m/s)
Katrina (2005)	8735180	0.94	−0.24	0.94	6.46
Katrina (2005)	8761724	0.97	0.05	0.89	16.07
Ike (2008)	8768094	0.74	−0.47	0.80	10.47
Ike (2008)	8771510	0.70	−0.17	0.73	15.31

## Usage Notes

This section highlights important considerations users should read before working with the hindcast archives. The dataset supports coastwide extreme-value analysis, ML-based model development, and provides boundary/forcing fields for higher-resolution local models, but users must account for several practical issues described below.

### Provided variables and file structure

Each decade archive (for example, AdcSwan_StormSurge_Data_2011_2021.nc) contains node coordinates (longitude(node), latitude(node)), a storm index (subdir_names), ISO-8601 formatted datetime strings (time_str, and the following model output variables:

- zeta - water-surface elevation (m, referenced to Model Zero datum)
- swan_HS - significant wave height (m)
- swan_TPS - peak wave period (s)

The time_str variable contains ISO-8601 formatted timestamps (YYYY-MM-DDTHH:MM:SS) for each hourly timestep, derived from the simulation start time recorded in the NHC best-track file (fort.22) and ADCIRC elapsed time in seconds. Each column of time_str corresponds to a unique storm case in subdir_names.

### Spatial domain and node coverage

Figure 7 shows the spatial coverage and distribution of model nodes from the mesh used in these hindcasts. Users must choose extraction locations that fall within the model node coverage shown in Figure 7. Points located outside the mesh/node will not return valid model data (extractions will be empty or NaN). To avoid this, verify that your target lon/lat lies within the node extents (or that the nearest model node is acceptably close) before extracting time series.

### Vertical datum and units

The model grid and bathymetry are referenced to the Model Zero (MZ) datum (see Szpilka *et al*.^30^). MZ approximates regional mean sea level but may differ from local tide gauge MSL by up to approximately 0.4 m in some places. In the Technical Validation, we applied location-specific vertical offsets when comparing to local gauges. Users extracting absolute elevations for local inundation studies should verify vertical references and, where necessary, apply site-specific corrections using the validation notes.

### Known limitations

- Wind forcing limitations: we used a parametric wind model, GAHM. Parametric forcings can underrepresent complex asymmetric wind structures and rapid structural evolution; these limitations propagate into wave and surge predictions. In particular, the GAHM represents only the hurricane vortex wind field and does not include large-scale synoptic meteorological winds, which can contribute to forerunner surge (a pre-landfall rise in water levels) several days before the storm center approaches the coast, as notably observed during Hurricane Ike (2008). Kennedy *et al*.^50^ showed that the Ike forerunner was generated by Ekman setup from persistent, large-scale, shore-parallel winds over the broad, shallow Texas–Louisiana shelf—a spatial wind footprint that a parametric vortex model cannot reproduce. Additionally, our model uses a uniform bottom friction coefficient (*C*_*f*_ = 0.0025), which may be too high to capture the Ekman-driven shelf-scale setup responsible for forerunner surge in the 2D barotropic framework; Pringle *et al*.^35^ similarly attributed a pre-landfall negative bias in Hurricane Katrina storm tide to insufficient forerunner generation in a depth-averaged model, and noted that using a smaller *C*_*f*_ on the continental shelf improved forerunner representation. Hope *et al*.^46^ demonstrated that accurately modeling the Ike forerunner required a specially tuned, very low bottom friction (Manning’s *n* = 0.012) for the muddy shelf and a data-assimilated wind product—neither of which is feasible for a globally consistent 232-storm database configuration.
- Depth-averaged current formulation: the 2D depth-integrated ADCIRC model does not resolve the vertical structure of currents. During hurricanes, surface currents driven by wind stress can differ significantly from the depth-averaged flow, which means SWAN receives depth-averaged rather than surface-layer currents. This may not fully capture the Doppler-shift and current-refraction effects on waves; Lin *et al*.^41^ demonstrated, using a fully 3D coupled ROMS–SWAN system, that explicitly resolving three-dimensional wave–current interactions significantly reduces the positive wave height bias relative to 2D-coupled configurations, providing a physics-based explanation for the systematic overestimation documented in the Technical Validation section. We also note that mesoscale baroclinic features such as the Loop Current in the Gulf of Mexico—which can further modify the wave field through wave-current interaction^40^—are not fully represented in a 2D barotropic framework. While a 3D baroclinic model would resolve density-driven vertical shear and mesoscale eddies more accurately, the computational cost for 232 events would be orders of magnitude higher without necessarily improving peak storm surge accuracy, which is primarily driven by barotropic wind-stress and pressure forcing in shallow coastal areas. The 2D ADCIRC+SWAN framework has been extensively validated for hurricane storm surge prediction^9,10,16,51^ and is used operationally by the NOAA Coastal Emergency Risk Assessment (CERA) platform^29^ and the U.S. Army Corps of Engineers for real-time and hindcast coastal flood guidance.
- No floodplain inundation: our mesh strictly follows the shoreline boundary and does not extend onto dry land or narrow channels and inlets. Without a floodplain for surge water to inundate, water is artificially confined to the wet domain, contributing to overestimation of peak coastal water levels as documented in our own validation (Table 2). Inundation over land dissipates surge energy and reduces peak coastal water levels; its omission is a known conservative bias in mesh configurations that do not include overland flooding^35^.
- Wave biases: validation shows systematic positive biases in significant wave height in deeper water (see Technical Validation). Users studying offshore wave extremes should treat wave values with caution. Additionally, we prescribe only tidal forcing at the eastern open boundary; remotely generated swells propagating into the domain from the open Atlantic are not captured, which may further contribute to wave discrepancies at offshore buoy locations.
- Mesh resolution is practical (nearshore approximately 100–500 m) but not suitable for building-level inundation or very small channels/inlets omitted from the mesh. Use local high-resolution models for detailed inundation mapping.

### Validation summary and expected skill

We validated water levels and wave metrics against numerous NOAA tide gauges^18^ and NDBC buoys^19^. Water-surface elevation shows high temporal skill (mean Pearson R  ≈ 0.86 across validation cases), while wave metrics exhibit larger regional variability and documented biases (see Technical Validation and accompanying plots). We advise reviewing the model results for the events and stations relevant to your work carefully before using this dataset for any critical decision-making.

### Quick example: access with Python/xarray

We provide a spatial visualization tool (extract_storm_spatial_map.py) in the dataset Zenodo directory (https://doi.org/10.5281/zenodo.17905131) that generates publication-quality maps showing the maximum water surface elevation across the entire computational domain for individual storm events. Users specify a storm identifier (e.g., bal122005 for Hurricane Katrina) and the NetCDF file path (respective decade file), and the script produces a 300-DPI georeferenced map with US coastlines, state boundaries, and a color-scaled representation of peak storm surge values.

We also provide an example script (extract_node_timeseries.py) for opening a decade file and extracting peak values of the variables at the node nearest a target lon/lat. A companion plotting script (extract_timeseries_all_files.py) extracts the time series at the same user-selected location and generates a three-panel figure showing the temporal evolution of water surface elevation (*ζ*), significant wave height (*H*_*s*_), and peak wave period (*T*_*p*_) across all storm events in a given decade file.

## Data Availability

ADCIRC (https://github.com/adcirc/adcirc) and SWAN (https://swanmodel.sourceforge.io/download/download.htm) are open source models. The coupled version used in this study can be found in the same ADCIRC github repository. We used OceanMesh2D tool (https://github.com/CHLNDDEV/OceanMesh2D) for pre-processing ADCIRC input files. The 2D color maps are produced using cmocean (https://github.com/matplotlib/cmocean). Custom Python scripts for data extraction and visualization (extract_storm_spatial_map.py, extract_node_timeseries.py, and extract_timeseries_all_files.py) are deposited alongside the dataset in the Zenodo repository (https://doi.org/10.5281/zenodo.17905131).
